# Quantification of proteome changes in bovine muscle from two-dimensional electrophoresis data

**DOI:** 10.1016/j.dib.2015.04.011

**Published:** 2015-05-07

**Authors:** Daniel Franco, Ariadna Mato, Francisco J. Salgado, María López-Pedrouso, Mónica Carrera, Susana Bravo, María Parrado, José M. Gallardo, Carlos Zapata

**Affiliations:** a Meat Technology Center of Galicia, r/Galicia 4, Parque Tecnolóxico de Galicia, San Cibrao das Viñas, Ourense 32900, Galicia, Spain; b Department of Genetics, University of Santiago de Compostela, Santiago de Compostela 15782, Spain; c Department of Biochemistry and Molecular Biology, University of Santiago de Compostela, Santiago de Compostela 15782, Spain; d Spanish National Research Council (CSIC), Marine Research Institute (IIM), Eduardo Cabello 6, Vigo 36208, Pontevedra, Spain; e Proteomics Laboratory, CHUS, Santiago de Compostela 15782, Spain

**Keywords:** Bovine muscle proteome, Stress-dependent proteome changes, Two-dimensional electrophoresis, Bootstrap resampling method, Relative and fold change measures, Quantitative proteomics

## Abstract

Proteome changes in the *longissimus thoracis* bovine muscle in response to pre-slaughter stress were assessed on the basis of two-dimensional electrophoresis (2-DE) data. In this study, the bootstrap resampling statistical technique and a new measure of relative change of the volume of 2-DE protein spots are shown to be more efficient than commonly used statistics to reliably quantify changes in protein abundance in stress response. The data are supplied in this article and are related to “Tackling proteome changes in the *longissimus thoracis* bovine muscle in response to pre-slaughter stress” by Franco et al. [1].

**Specifications table**

**Table:** 

Subject area	Biology
More specific subject area	Meat science, bovine muscle proteome
Type of data	Table, figure
How data was acquired	2-DE gel images stained for total protein with SYPRO Ruby stain were acquired using a Gel Doc XR+system. Image analysis of digitalized gels was performed through PDQuest Advanced software
Data format	Analyzed output data
Experimental factors	2-DE was performed using total protein extracts from normal meat and DFD meat of animals affected by pre-slaughter stress
Experimental features	2-DE gels (1st dimension pH 4-7 gradient; 2nd dimension 12% SDS-PAGE) were obtained, protein spots were excised and peptides were analyzed after trypsin digestion by LC-MS/MS and MALDI-TOF/TOF MS
Data source location	Lugo, Spain
Data accessibility	Data are with this article and provided in Supplementary materials directly with this article

Value of the data

• Proteome changes in bovine muscle in response to pre-slaughter stress using 2-DE data.
• Application of the bootstrap statistical technique for testing proteome changes in cattle.
• Illustration of the efficiency of “relative change” as a new measure in quantitative proteomics.

## 1 Experimental design

Valuable information about the proteome changes in the *longissimus thoracis* (LT) bovine muscle in response to pre-slaughter stress (PSS) was obtained from 2-DE data. The occurrence of Dark, Firm and Dry (DFD) meat was used as indicator of animals affected by PSS. A total of four biological replicates of control (normal or non-DFD) and DFD meats from male calves of the Rubia Gallega breed (Spain) were used in this study. DFD and control samples were selected from 76 male calves after evaluation of meat quality parameters that differentiate both types of meat [1]. 2-DE protein spots with statistically significant changes in protein abundance between control and DFD samples were identified by mass spectrometry (MS).

## 2 Materials and methods

### 2.1 Meat sample preparation, protein extraction and quantification

Meat samples from the LT bovine muscle were excised from the left half of each carcass at 24 h post-mortem. A 2.5 cm thick steak was taken at the fifth rib and packed under vacuum conditions at the abattoir and subsequently transported to laboratory under refrigerated conditions. Meat samples were then lyophilized under optimal conditions [2] and frozen at −80 °C until proteome analysis. Lyophilization is a cheap, practical and safe alternative for the storage of samples prior to protein extraction, separation and quantification by 2-DE [2].

Lyophilized beef powder (50 mg) was resuspended in 1.5 mL of lysis buffer (7 M urea; 2 M thiourea; 4% CHAPS; 10 mM DTT; and 2% pharmalyte pH 3-10, GE Healthcare) for 2 h at 25 °C. An aliquot of 250 µL was lysed using a Sonifier 250 (Branson) by cycling. Protein purification and extraction from crude cell lysates were carried out with the Clean-Up kit (GE Healthcare) as described in manufacturer׳s indications. The proteins were then resuspended in 250 µL of lysis buffer. Protein quantification was assessed for each extraction using the CB-X protein assay kit (G-Biosciences) according to manufacturer׳s recommendations. The BSA protein standard was used to get a calibration curve.

### 2.2 Two- dimensional electrophoresis

2-DE was carried out from 350 μg of total protein extract dissolved in lysis and rehydration (7 M urea, 2 M thiourea, 4% CHAPS, 0.002% bromophenol blue) buffers. Protein extracts were loaded into 24-cm-long ReadyStrip IPG Strips (Bio-Rad Laboratories) with linear pH gradient of 4-7, together with 0.6% DTT and 1% IPG buffer (Bio-Rad Laboratories). First dimensional isoelectric focusing (IEF) was performed using a PROTEAN IEF cell system (Bio-Rad Laboratories). Gels were initially rehydrated for 12 h at 50 V and rapid voltage ramping was subsequently applied to reach a total of 70 kVh. After IEF, strips were equilibrated for 15 min at room temperature in the equilibration solution I (50 mM Tris pH 8.8, 6 M urea, 2% SDS, 30% glycerol and 1% DTT) and then with equilibration solution II (50 mM Tris pH 8.8, 6 M urea, 2% SDS, 30% glycerol and 4% iodoacetamide) under the same conditions. Second dimension electrophoresis was run on 12% SDS-PAGE gels using an Ettan DALTsix vertical slab gel system (GE, Healthcare) and Tris-glycine-SDS (50 mM Tris, 384 mM glycine and 0.2% SDS) as electrode buffer. Gels were run at a constant current of 32 mA at 25 °C.

### 2.3 Image analysis

2-DE gel images stained for total protein with SYPRO Ruby stain (Lonza) were acquired using a Gel Doc XR+ system (Bio-Rad Laboratories). Image analysis of digitalized gels was performed through PDQuest Advanced software v. 8.0.1 (Bio-Rad Laboratories). 2-DE gels were matched across biological replicates and volume of each spot was quantitatively determined after background subtraction and normalization using total density of validated spots across all replicate gels. The pI and M_r_ of spots were determined from their position on the IEF-strips and standard molecular mass markers ranging from 15 to 200 kDa (Fermentas), respectively.

Sample lyophilization, protein extraction and 2-DE methods used in the study resulted in good quality and highly reproducible 2-DE gel images (Supplementary Fig. 1).

### 2.4 Mass spectrometry analysis

A total of ten 2-DE protein spots showed statistically significant differential abundance in muscle conversion to DFD meat [1]. Differentially abundant protein spots were excised and peptides were analyzed after trypsin digestion by LC-MS/MS and MALDI-TOF/TOF MS as described in Franco et al. [1]. The proteins identified by MS were: myosin light chain 3 (MYL3), myosin light chain 6B (MYL6B), myosin regulatory light chain 2 (MYL2), troponin C type 2 (TNNC2), beta-galactoside alpha-2,6-syalyltransferase 1 (ST6GAL1), ATP synthase subunit beta (ATP5B), triosephosphate isomerase (TPI1), cofilin-2 (CFL2) and two fast skeletal myosin regulatory light chain 2 isoforms (MYLPF and MYLPF-1) (Supplementary Tables 1 and 2).

### 2.5 Statistical analysis

Statistically significant differential abundance between control and DFD groups was assessed by bootstrap resampling. Bootstrapping was used to calculate 95% and 99% non-parametric confidence intervals (CIs) for the means of the observed spot volumes in DFD and control biological replicates by the bias-corrected percentile method [3]. For each set of *N* (=4) estimates of spot volume, 2000 bootstrap samples of size *N* were drawn with replacement following a Monte Carlo algorithm. Random numbers were obtained using the standard multiplicative linear congruential generator implemented by Schrage [4], which uses the multiplier 16807 and prime modulus 2^31^−1 to generate very long sequences of “pseudo-random” numbers that have the appearance of randomness. Bootstrap CIs (95 and 99%) were then constructed from distribution of 2000 bootstrap mean replications. The bias was corrected from the proportion of bootstrap mean replications less than the original estimate of the mean using the theoretical normal distribution [3]. The bootstrapped CIs were calculated with the software DIANA [6].

The bootstrap-based statistical approach offers clear advantages in comparison with commonly used tests for testing differential protein abundance between samples: Student׳s *t*-test and Mann–Whitney *U*-test [3,5]. The bootstrap algorithm uses the empirical distribution for the sample mean. In contrast, the *t*-test is based on the *t*-distribution (small samples) or normal distribution (large samples), which are parametric and theoretical distributions that are generally only approached by the sample data. On the other hand, the Mann–Whitney is a non-parametric test but it cannot provide estimates of the magnitude of any difference because the observed data values are replaced by their ranks.

Biased volume distributions of 2-DE protein spots have a marked negative effect on the efficiency of the Student׳s *t*-test and the Mann–Whitney *U*-test. Bias of mean volume (×10^−2^) averaged across biological replicates for each 2-DE protein spot with significant differential abundance between control and DFD samples is shown in Table 1. The bias for each spot was estimated as the percentage of 2000 bootstrap mean replications with mean volume less than the observed mean volume that deviates from expected percentage of 50% for unbiased distributions. It can be seen that empirical distributions of mean volumes were highly biased for an important number of the identified proteins in PSS response, with biases up to 50% (Table 1). It is noteworthy that the sampling distribution of the means of random samples of any distribution will approach the normal distribution when the sample size is sufficiently large, according with the central limit theorem [5]. However, small replicate numbers are commonly used in proteomic studies.

### 2.6 Measures to quantify changes in protein abundance

The commonly used measure of “fold change” (FC) was applied to quantify volume changes of 2-DE protein spots from control to DFD meat as follows:

$$\mathrm{FC}={V_{\mathrm{DFD}}/V}_{C}$$

where $V_{\mathrm{DFD}}$ and $V_{C}$ are the volume means for each spot across replicate gels in DFD and control samples, respectively. FC-values less than one were represented as their negative reciprocal. In this study, a new measure is used for determination of quantitative changes in protein abundance between groups. The measure named “relative change” (RC) is defined as follows:

$$\text{RC}=\mathrm{DV}/\left|{\mathrm{DV}}_{\max}\right|$$

where $\mathrm{DV}=V_{\mathrm{DFD}}-V_{C}$ is a measure of the differential volume between samples, and ${\mathrm{DV}}_{\max}$ is the maximum observed value of DV over spots.

**Fig. 1:**
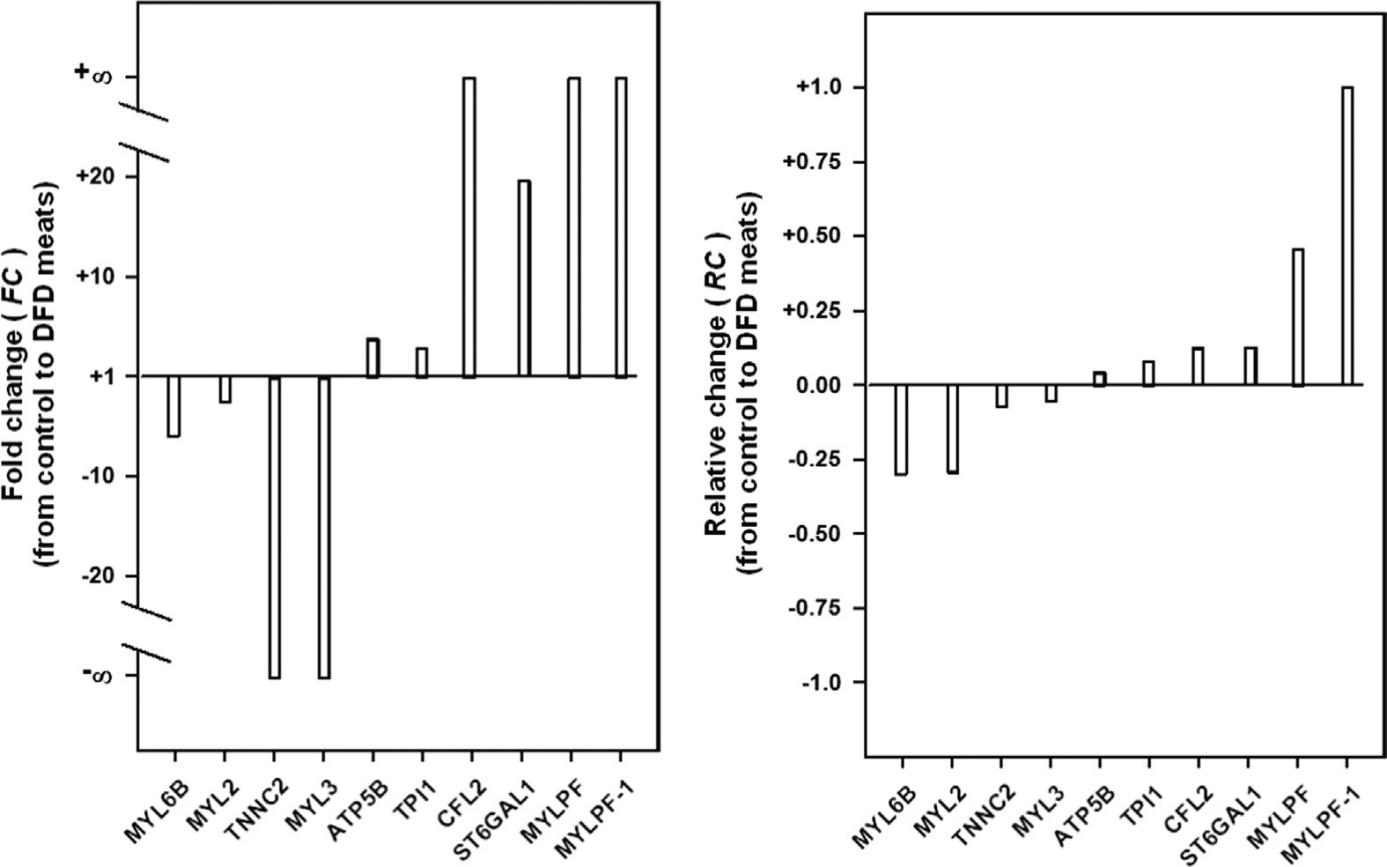
Fold change (FC) and relative change (RC) in the volume of 2-DE protein spots.

The RC measure provides more genuine information than FC to assess differential protein abundance between groups. By way of illustration, changes in protein abundance between control and DFD meat samples as measured by FC and RC *s*tatistics are shown in Fig. 1. It can be seen that both measures provide very discrepant estimates of the strength of change in protein abundance from control to DFD samples. However, *RC* outperforms *FC* to assess volume changes because FC ranges from −∞ to +∞ while RC ranges between −1.0 and +1.0 and takes a value of zero when there is no volume change between groups. Note that the FC measure gives values of −∞/+∞ for qualitative changes in protein abundance (i.e., presence or absence of a given spot in only one of the sample groups) when the absence of a given spot can be due merely to the occurrence of protein amounts undetectable by 2-DE. In contrast, RC allows us the joint analysis of spots with qualitative and quantitative changes under the same range of variation. Overall, the RC measure enables to assess more exactly the relative strength (weak, moderate or strong) of change in protein abundance between sample groups.

## Figures and Tables

**Table 1:** Biases of mean volumes for differentially abundant protein spots between control and DFD meat samples.

Protein (Abbrev.)	Control meat		DFD meat	
	Mean volume	Bias	Mean volume	Bias
	(±SE, standard error)	(%)	(±SE, standard error)	(%)
MYL3	2.16±0.46	0.6	0.00	N/A
MYL6B	13.6±8.41	17.2	2.40±0.71	10.6
MYL2	18.7±3.24	6.2	7.81±1.83	9.4
TNNC2	2.27±0.94	8.0	0.00	N/A
ST6GAL1	0.28±0.28	49.2	5.60±3.31	51.0
ATP5B	0.62±0.19	11.2	2.35±0.49	7.6
TPI1	3.03±0.12	6.0	6.83±2.61	10.2
CFL2	0.00	N/A	4.72±1.34	11.4
MYLPF	0.00	N/A	18.1±6.15	1.0
MYLPF-1	0.00	N/A	38.1±14.5	4.6

## Footnotes

Appendix A Supplementary data associated with this article can be found in the online version at doi:10.1016/j.dib.2015.04.011.
