# Supplementary material for: Quantification of proteome changes in bovine muscle from two-dimensional electrophoresis data
Source: Data Brief. 2015 May 7;4:100–4. doi: 10.1016/j.dib.2015.04.011 (PMC4510394; doi:10.1016/j.dib.2015.04.011)
Supplement: Supplementary file 1 — Supplementary material [file mmc1.zip › Supplementary Table 2.doc]

| Supplementary Table 2 - Identification of protein spots differentially abundant in control and DFD meat samples from the *longissimus* *thoracis* bovine muscle by MALDI TOF/TOF MS. | | | | | |
| --- | --- | --- | --- | --- | --- |
| Spot  code^a^ | Protein name | Mascot score | Match./  Cov.(%)^b^ | Peptides | |
|  |  |  |  | [M+H] | Sequence |
| C5 | Myosin light chain 3 (MYL3) | 456 | 8/56 | 998.38 | EAFTLFDR |
|  |  |  |  | 1249.44 | EGNGTVMGAELR |
|  |  |  |  | 1281.49 | ITYGQCGDVLR |
|  |  |  |  | 1396.61 | ALGQNPTQAEVLR |
|  |  |  |  | 1501.53 | DTGTYEDFVEGLR |
|  |  |  |  | 1509.59 | IEFTPEQIEEFK |
|  |  |  |  | 2001.70 | MMDFDTFLPMLQHISK |
|  |  |  |  | 2637.06 | AAAAPAPAPAPPPAPEPSKEPEFDPSK |
| C6 | Myosin light chain 6B, alkali, smooth muscle and non-muscle (MYL6B) | 349 | 9/47 | 791.42 | VMGAELR |
|  |  |  |  | 1025.59 | HVLTTLGER |
|  |  |  |  | 1042.51 | EAFELYDR |
|  |  |  |  | 1139.66 | IQEPPIDLSK |
|  |  |  |  | 1300.65 | GSYQDYLEGLR |
|  |  |  |  | 1356.64 | IQFSQCGDVMR |
|  |  |  |  | 1382.78 | ALGQNPTNAEVLR |
|  |  |  |  | 1724.93 | VDFETFLPMLQAVAK |
|  |  |  |  | 1746.12 | VELPSLIPVILEKPAK |
| C9 | Myosin regulatory light chain 2, ventricular/cardiac muscle isoform (MYL2) | 166 | 8/40 | 791.29 | VFDPEGK |
|  |  |  |  | 850.33 | DTFAALGR |
|  |  |  |  | 1094.38 | EMLTTQAER |
|  |  |  |  | 1136.38 | NEEIDEMLK |
|  |  |  |  | 1192.45 | DGFIDKNDLR |
|  |  |  |  | 1240.42 | EAFTIMDQNR |
|  |  |  |  | 1404.53 | GADPEETILNAFK |
|  |  |  |  | 1645.67 | LKGADPEETILNAFK |
| C11 | Unidentified |  |  |  |  |
| D1 | Unidentified |  |  |  |  |
| D2 | ATP synthase subunit beta, mitochondrial  (ATP5B) | 299 | 9/24 | 1038.47 | IPVGPETLGR |
|  |  |  |  | 1401.55 | IMNVIGEPIDER |
|  |  |  |  | 1406.52 | AHGGYSVFAGVGER |
|  |  |  |  | 1435.60 | FTQAGSEVSALLGR |
|  |  |  |  | 1439.62 | VALTGLTVAEYFR |
|  |  |  |  | 1617.62 | VALVYGQMNEPPGAR |
|  |  |  |  | 1650.74 | LVLEVAQHLGESTVR |
|  |  |  |  | 1831.68 | IMDPNIVGSEHYDVAR |
|  |  |  |  | 1987.82 | AIAELGIYPAVDPLDSTSR |
| D3 | Triosephosphate isomerase (TPI1) | 318 | 14/67 | 747.31 | EAGITEK |
|  |  |  |  | 758.37 | VIADNVK |
|  |  |  |  | 850.38 | VVFEQTK |
|  |  |  |  | 954.39 | FFVGGNWK |
|  |  |  |  | 1137.46 | IAVAAQNCYK |
|  |  |  |  | 1204.48 | SNVSDAVAQSAR |
|  |  |  |  | 1326.55 | IIYGGSVTGATCK |
|  |  |  |  | 1458.59 | HVFGESDELIGQK |
|  |  |  |  | 1466.59 | TATPQQAQEVHEK |
|  |  |  |  | 1484.67 | NNLGELINTLNAAK |
|  |  |  |  | 1539.64 | DLGATWVVLGHSER |
|  |  |  |  | 1602.71 | VVLAYEPVWAIGTGK |
|  |  |  |  | 1607.66 | VANGAFTGEISPGMIK |
|  |  |  |  | 2191.87 | VPADTEVVCAPPTAYIDFAR |
| D5 | Cofilin-2 (CFL2) | 80 | 5/29 | 1000.70 | LLPLNDCR |
|  |  |  |  | 1024.70 | VFNDMKVR |
|  |  |  |  | 1322.85 | MIYASSKDAIK |
|  |  |  |  | 1323.91 | AVLFCLSDDKR |
|  |  |  |  | 1337.85 | YALYDATYETK |
| D6 | Myosin regulatory light chain 2, skeletal muscle  isoform (MYLPF) | 298 | 9/60 | 880.37 | FSQEEIK |
|  |  |  |  | 884.31 | DTFAAMGR |
|  |  |  |  | 1173.52 | DGIIDKEDLR |
|  |  |  |  | 1192.50 | EAFTVIDQNR |
|  |  |  |  | 1319.54 | GADPEDVITGAFK |
|  |  |  |  | 1524.62 | FLEELLTTQCDR |
|  |  |  |  | 2016.82 | EASGPINFTVFLNMFGEK |
|  |  |  |  | 2480.92 | AAAEGGSSSVFSMFDQTQIQEFK |
|  |  |  |  | 2716.86 | RAAAEGGSSSVFSMFDQTQIQEFK |
| D7 | Myosin regulatory light chain 2, skeletal muscle  isoform (MYLPF-1) | 237 | 8/56 | 884.30 | DTFAAMGR |
|  |  |  |  | 1173.51 | DGIIDKEDLR |
|  |  |  |  | 1192.49 | EAFTVIDQNR |
|  |  |  |  | 1319.52 | GADPEDVITGAFK |
|  |  |  |  | 1524.60 | FLEELLTTQCDR |
|  |  |  |  | 1560.67 | LKGADPEDVITGAFK |
|  |  |  |  | 2016.80 | EASGPINFTVFLNMFGEK |
|  |  |  |  | 2480.92 | AAAEGGSSSVFSMFDQTQIQEFK |
| ^a^ Gel position of assigned spots is shown in Fig. 1 [1]; ^b^ Matched peptides and percentage of the polypeptide sequence covered by matched peptides. | | | | | |
