# Supplementary material for: Quantification of proteome changes in bovine muscle from two-dimensional electrophoresis data
Source: Data Brief. 2015 May 7;4:100–4. doi: 10.1016/j.dib.2015.04.011 (PMC4510394; doi:10.1016/j.dib.2015.04.011)
Supplement: Supplementary file 1 — Supplementary material [file mmc1.zip › Supplementary Figure 1.doc]

Supplementary Fig 1 - 2-DE protein profiles obtained from two technical replicates of normal (or control) meat samples of the *longissimus* *thoracis* bovine muscle (Rubia Gallega breed).
